# Clinical findings in congenital infection by Zika virus: a retrospective study in a reference hospital in Central-West Brazil

**DOI:** 10.1186/s12887-019-1762-6

**Published:** 2019-10-29

**Authors:** Cláudia de Paula Guimarães, Myrella Silveira Macedo, Maria Alves Barbosa, Solomar Martins Marques, Paulo Sucasas Costa, Ênio Chaves de Oliveira

**Affiliations:** 1 Hospital das Clínicas da UFG/EBSERH, Goiânia, GO Brazil; 2 Secretaria Municipal de Saúde, Goiânia, GO Brazil; 3 Faculdade de Enfermagem da UFG, Goiânia, GO Brazil; 4 Faculdade de Medicina da UFG, Goiânia, GO Brazil

**Keywords:** Zika virus, Microcephaly, Malformations

## Abstract

**Background:**

An increased number of congenital Zika virus infections with neurological and musculoskeletal malformations have been diagnosed worldwide, however, there are still several gaps in the knowledge about this infection, its associated mechanism, timing of transmission, and description of throughout findings of signs and symptoms, which is described in this paper. The purpose of this study is to describe aspects of congenital Zika syndrome (CZS) beyond the central nervous system comprising detailed delineation of all the other clinical findings.

**Methods:**

A retrospective research developed using electronic medical records. We analyzed the files of 69 children with an initial diagnosis of microcephaly by Zika vírus who were born in 2015, 2016 and 2017, treated during the period from 2016 to 2017.

**Results:**

The newborns presented several neurological and musculoskeletal malformations, eye damage, hearing impairment and other malformations.

**Conclusions:**

The present study has significant impact for health care teams following lactents with Congenital Zika Syndrome.

## Introduction

Zika virus (ZIKV) is an Arbovírus, belonging to the genera Flavivirus (Flaviviridadae family) which is transmitted primarily via *Aedes aegypti*. In 2015, The World Health Organization (WHO) recognized that Zika virus was responsible for microcephaly and related neurologic disorders in infants born from infected mothers [1].

The vertical transmission’s mechanism of ZIKV can occur in different gestational ages [2], even by breastfeeding [3], since the placenta acts on one hand as the only physical and immunological barrier for the fetus and on the other hand as potential viral reservoir [4].

Therefore, the purpose of this study is to describe aspects of congenital Zika syndrome (CZS) beyond the central nervous system comprising detailed delineation of all the other clinical findings.

## Methods

This is a retrospective research, developed using electronic medical records. The study was carried out in the city of Goiania-GO, Brazil.

As most infants were included and investigated at the beginning of the Zika virus epidemic, microcephaly was the main clinical marker and was used as the inclusion criterion. Thus, in this study the inclusion criterion was an initial diagnosis of microcephaly. We identified 233 medical records with CID 10 Q02, after thorough data analysis identified 69 cases of microcephaly by Zika virus from newborns who were born in 2015, 2016 and 2017 and treated during the period from 2016 to 2017 in CRER - Rehabilitation and Recover Center Dr. Henrique Santillo.

Diagnostic criteria for Zika virus infection were performed by serology (IgM, IgG), and molecular tests (PCR +); and neuroimaging examinations (MRI and CT) of infants that confirmed the pattern of sequelae of ZIKV infection.

There was no serological and / or molecular diagnosis in all cases during pregnancy, so the definitive diagnosis was also made through postnatal neuroimaging examinations in newborns.

Data were analyzed by Statistical Package for the Social Sciences - SPSS 20.0.

## Results

During the study period, there was a total of 233 infants with microcephaly at the outpatient clinic, and 164 were excluded because they were microcephaly for other causes. After analyzing the medical records, we identified: 04 cases of hypoxia, 07 prematurity, 37 CP (cerebral palsy), 17 congenital toxoplasmosis, 12 SGA (small for gestational age), 08 congenital cytomegalovirus, 07 CNS malformation, 04 IUGR (intrauterine growth retardation), 04 congenital syphilis, 03 congenital rubella, 02 Down’s syndromes, 02 neurodevelopment delay, 20 genetic causes, and 37 microcephaly from other causes.

During pregnancy 41 women who had Zika infection documented by serology (IgM +), and / or molecular tests (PCR +). Neuroimaging examinations of newborns, such as computed tomography and nuclear magnetic resonance, were performed in 28 infants and confirmed the pattern of sequelae of ZIKV infection.

Thus, 69 records of lactents with microcephaly infected by CZS were analyzed, of which 41 (59.4%) were female; 6 were born in 2015, 55 in 2016 and 8 in 2017. Microcephaly was detected in the intrauterine period in 24 of the fetuses, by performing morphological ultrasonography, 43 diagnosed after birth (2 missing data).

Regarding gestational age, there were 14 preterm newborns, 52 term and 3 missing data; 21 SGA (small for gestational age), 46 AGA (appropriate for gestational age), 1 LGA (large for gestational age), 1 missing data and one twin (only one fetus was infected). The cephalic perimeter of the newborn at birth measured an average of 29.44 cm (ranging from 22 to 37 cm), the length 45.83 cm and the weight was 2592.88 g.

**Fig. 1:**
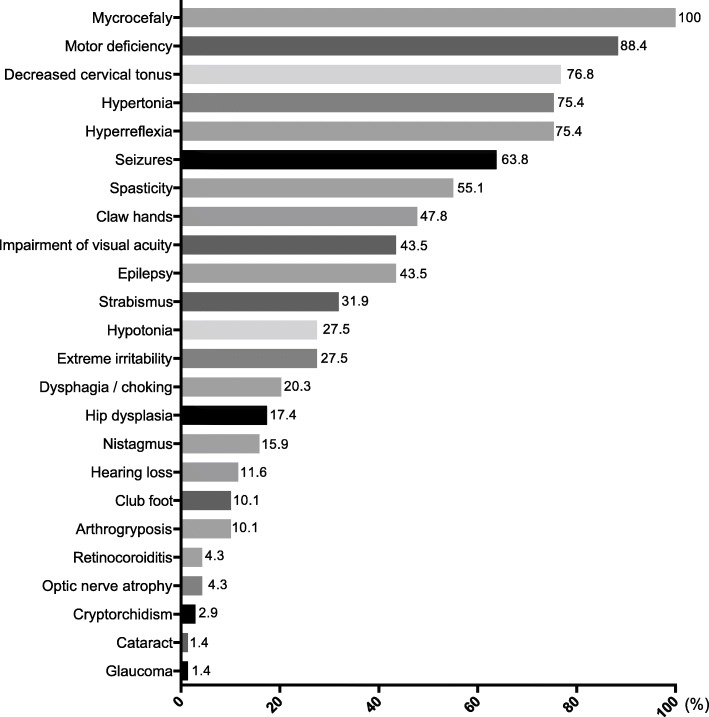
Distribution of clinical finding of Zika virus congenital syndrome

The clinical data of infants are detailed in Fig. 1.

## Dicussion

In this study, the diagnosis of Zika virus infection in most pregnant women was made by serological and molecular tests. In other pregnant women with clinically confirmed Zika infection but who did not have laboratory tests, neuroimaging examinations of infants confirmed the pattern of sequelae of ZIKV infection. In another study of 345 women from September 2015 to May 2016, 182 (53%) women were infected with ZIKV between 6 and 39 weeks of gestation and had positive ZIKV results in blood, urine or both. The correlation of ZIKV infection with CZS and microcephaly has been demonstrated [5, 6].

This study demonstrated that CZS can cause a constellation of clinical malformations in addition to the central nervous system (classically microcephaly), with special emphasis on motor alterations (mainly motor deficiency, hyper and hypotonia, hyperreflexia, hip dysplasia and arthrogryposis), ophthalmologic (P.E. impairment of visual acuity, glaucoma, retinocoroiditis and optic nerve atrophy) and others (cryptorchidism, dysphagia / choking). In addition, other studies have identified neurological abnormalities and musculoskeletal malformations similar to our findings [7, 8].

A total of 24 newborns were diagnosed with CZS during gestation, through the morphological USG that identified the microcephaly intrafetal. Of these, 15 also confirmed the diagnosis of maternal Zika virus infection by serology (IgM) and / or molecular tests (PCR). Nine of these infants had the pattern of sequelae of ZIKV infection confirmed by neuroimaging (MRI and CT). Microcephaly findings on intrauterine ultrasonography were also observed in a similar study and endorse our data [7].

As a result, the relationship of ZIKV infection to fetal brain development is poorly understood, as well as whether the risk of malformations is restricted to transmission of infection during the first trimester of pregnancy. Experimental study in animals demonstrated a high and selective tropism by brain and nerve cells promoting various types of lesions [9]. Despite the impact of malformations, especially microcephaly, there is a concern to overestimate the diagnosis of this condition and increase anxiety in potentially infected pregnant women and the risk of interruption of pregnancy in healthy fetuses [10].

In this study, it was observed that, of the 233 cases of infants with microcephaly, 69 of these were consequences of CZS, there was the identification of ZIKV infection in 29.6% of children affected in the period between 2015 and 2017, with the highest number of cases occurred in the period of 2016, most females, term, AGA (adequate for gestational age) and two twins (with only one of the fetuses with sequelae of infection by Zika virus).

Other clinical aspects besides microcephaly have been highlighted in the literature, such as the occurrence of epilepsy. Our data revealed a total of 43.5% of lactents with epilepsy, consistent with recent study [11], in which a total of 67% epilepsy was observed in 141 newborns with CZS, more than a half (56%) requiring anticonvulsive polytherapy, highlighting the severity of congenital lesions by ZIKV.

The World Health Organization (WHO) standardized the references of the head circumference (HC) measurement for the diagnosis of microcephaly by CZS, namely HC less than 2 standard deviations (HC < -2DP) according to gender and gestational age at birth. Thus, equal or less than 31.9 cm for boys and equal to or less than 31.5 cm for girls; for newborns with 37 weeks or more of gestation [12]. Thus, the values obtained in this study, whose mean of newborns head circumference was 29.44 cm on average, the length 45.83 cm and the weight 2592.88 g. In addition, another study revealed that head circumference at birth of newborns had an average of 29 cm [12], endorsing the measure of the head circumference observed in the records of the newborns of this study.

Another study described cases of twin pregnancies exposed to ZIKV where only one fetus was affected with microcephaly associated with severe damage to the central nervous system. It has also been explained that neural stem cells from different individuals could behave differently under ZIKV infection and that microcephaly is probably associated with abnormal cell function [13]. These data endorse the findings of this study which identified a case of twinhood where one fetus was infected by Zika virus with microcephaly and the other fetus was born without malformations.

Autopsy of fetuses of women infected by Zika virus identified, in addition to microcephaly and various CNS changes, abnormalities in the upper and lower limbs, congenital arthrogryposis, hip malformations, hypospadias, cryptorchidism, increased muscle tone, hyperreflexia, pulmonary hypoplasia and others congenital defects [14]. As compatible with our data, we observed that congenital microcephaly by the Zika virus can occur concomitantly with several malformations, namely: limb hypertonia and / or hypotonia, spasticity, clawed hands, flexoadducted thumbs, exalted reflexes (muscle stretching), absence of cephalic and cervical tonus, absence of trunk tonus, hyperreflexia, equine / club feet, hip malformation, arthrogryposis and cryptorchidism.

Other manifestations such as epilepsy, seizures, irritability, dysphagia, gait impairment due to motor deficiencies and decreased auditory acuity were identified in this study. Supporting our findings, other data revealed that some newborns had dysphagia, hearing and visual loss, as well as neurological abnormalities, hypertonia, hyperreflexia, irritability, tremors and seizures [15].

Ocular lesions were identified in lactents with microcephaly born from the Zika virus epidemic, for example: macular alterations with thick patches of retinal pigmentation and/or chorioretinal atrophy, hypoplasia, pallor and other abnormalities of the optic nerve, presence of macular pigmentation, neuroretinal macular atrophy, and loss of ocular reflex [16]. We identified optic nerve atrophy, cataracts, glaucoma, macular scars and cases of reticoroiditis (Roth spot). Other ophthalmologic changes were diagnosed in newborns, such as nystagmus and strabismus (convergent or divergent) and changes in visual acuity.

A weak point of our study is that patients are allocated based on the diagnosis of microcephaly. Recent reviews have shown that CZS can occur without the presence of microcephaly, in addition to the fact that microcephaly is not the most prevalent malformation [17]. A case-control study estimated that 2 to 5 newborns per 1000 livebirths may have microcephaly associated with the ZIKV epidemic [18].

However, when using the microcephaly inclusion criterion in CZS, the most severe cases were certainly included, which allows to map the associated manifestations of CSZ in a more comprehensive way.

## Conclusion

This study demonstrated that congenital Zika virus infection can occur with several distinct malformations and alterations that are aggravated by cognitive and motor deficits, epilepsy, difficulty in swallowing, visual and auditory system alterations and decreased and / or lost mobility.

These findings attest to the severity of the sequelae of this infection and strengthen the need to establish public policies to increase the knowledge and preparation of health professionals who have the challenge of facing such epidemics. However, mainly contribute to the adequate diagnosis of congenital Zika virus infection (with or without microcephaly) to provide the best development of infants with different deficits and malformations.

## Data Availability

The raw data will not be shared, because is also used for still unpublished work.

## Abbreviations

CEP: Research Ethics Committee
CNS: Central nervous system
CRER: Rehabilitation and Recovery Center Dr. Henrique Santillo
CZS: Congenital Zika Syndrome
EBSERH: Empresa Brasileira de Serviços Hospitalares
HC: Head circumference
LGA: Large for gestational age
NHC: National Health Council
SGA: Small for gestational age
SPSS20.0: Statical Package for the Social Sciences version 20.0
UFG: Universidade Federal de Goiás, Brazil
WHO: World Health Organization
ZIKV: Zika vírus
